# Effects of V Addition on the Deformation Mechanism and Mechanical Properties of Non-Equiatomic CoCrNi Medium-Entropy Alloys

**DOI:** 10.3390/ma16145167

**Published:** 2023-07-22

**Authors:** Rui Shen, Zengyu Ni, Siyuan Peng, Haile Yan, Yanzhong Tian

**Affiliations:** 1 Key Laboratory for Anisotropy and Texture of Materials (Ministry of Education), School of Materials Science and Engineering, Northeastern University, Shenyang 110819, China; 2000574@stu.neu.edu.cn (R.S.); 2200601@stu.neu.edu.cn (Z.N.); 2110165@stu.neu.edu.cn (S.P.); 2 Research Center for Metallic Wires, Northeastern University, Shenyang 110819, China

**Keywords:** medium-entropy alloy, stacking fault energy, transformation-induced plasticity, stacking fault, deformation mechanism

## Abstract

Equiatomic CoCrNi medium-entropy alloys exhibit superior strength and ductility. In this work, a non-equiatomic CoCrNi alloy with low stacking fault energy was designed, and different fractions of V were added to control the stacking fault energy and lattice distortion. Mechanical properties were evaluated by tensile tests, and deformation microstructures were characterized by transmission electron microscope (TEM). The main deformation mechanisms of CoCrNiV alloy with low V content are dislocation slip, stacking faults, and deformation-induced HCP phase transformation, while the dominant deformation patterns of CoCrNiV alloy with high V contents are dislocation slip and stacking faults. The yield strength increases dramatically when the V content is high, and the strain-hardening behavior changes non-monotonically with increasing the V content. V addition increases the stacking fault energy (SFE) and lattice distortion. The lower strain-hardening rate of 6V alloy than that of 2V alloy is dominated by the SFE. The higher strain-hardening rate of 10V alloy than that of 6V alloy is dominated by the lattice distortion. The effects of V addition on the SFE, lattice distortion, and strain-hardening behavior are discussed.

## 1. Introduction

Multi-principal element alloys (MPEAs) are of great interest to researchers because of their high compositional variability and multiple deformation mechanisms, which enable them to have excellent properties, including high strength, wear resistance, and energy storage capability [1,2,3,4,5]. Single-phase face-centered cubic (FCC) high/medium entropy alloys (HEAs/MEAs) have been extensively studied because of their excellent plasticity and good processing properties. HEAs/MEAs such as FeMnCoCr, CoCrNi, and FeCoCrNi usually undergo twinning deformation. The martensitic transformation from FCC to hexagonal close-packed (HCP) structure may occur under special temperatures and strain rates [6,7]. Various deformation mechanisms such as twinning-induced plasticity (TWIP) [8], transformation-induced plasticity (TRIP) [9], and stacking-fault-induced plasticity [10] can simultaneously improve the strength and ductility of MPEAs with FCC structures.

Among single-phase HEAs/MEAs, CoCrNi MEAs with single-phase FCC structure have extraordinary tensile properties and fracture toughness due to their low stacking fault energy (SFE) and high solid-solution strengthening effect [8,11,12,13]. Sohn et al. [14] found that the yield strength (*σ*_Y_) of FCC VCoNi alloy reaches 1 GPa (*d* = 2.0 ± 1.5 μm) through high solid-solution strengthening and grain boundary strengthening effects and that the nanoscale dislocation substructure gives the alloy excellent ductility and strain-hardening capability. Therefore, the solid-solution strengthening plays an important role in improving the yield strength of HEAs/MEAs and is associated with the following factors: the interaction energy between fluctuations in local solute concentration and dislocations [15], the charge transfer of atomic size and electronic states [16] and short-range ordering (SRO) or clustering (SRC) [17]. It was shown that the presence of large atomic volume misfit of V in CoCrNi alloys produced a high solid-solution strengthening effect, which increased the yield strength of FCC and body-centered cubic (BCC) HEAs [15].

Strain-hardening behavior, tensile strength, and ductility of the alloy are significantly affected by the plastic deformation mechanism, which is generally associated with SFE [18]. CoCrNi alloys have low SFE and exhibit deformation-induced twinning behavior. Our recent study has indicated that the change in the chemical composition of the ternary CoCrNi alloys can impact dramatically on the SFE and related deformation mechanisms [19]. In contrast, VCoNi alloys have relatively high SFE and exhibit dislocation planar slip behavior during deformation, resulting in high strength and excellent strain-hardening capability [8,11,14]. This is supposed to be induced by the severe lattice distortions due to V addition.

However, there are few studies on the effect of V addition on the deformation mechanism of non-equiatomic CoCrNi alloy. In this study, we change the V content of non-equiatomic CoCrNi alloys and comprehensively investigate the effects of SFE and solid solution of V on the deformation mechanism and mechanical behavior of the alloy in order to achieve excellent strength and strain-hardening capability. The proposed strategy provides a new solution for optimizing comprehensive mechanical properties and is expected to optimize the excellent mechanical properties of MPEAs in different systems.

## 2. Materials and Methods

Three types of alloys were prepared using raw materials with purity of 99.99 wt.%. Alloys Co_36_Cr_36_Ni_26_V_2_, Co_35_Cr_35_Ni_24_V_6_, Co_33_Cr_33_Ni_24_V_10_ were denoted as 2V, 6V and 10V, respectively. These ingots were prepared by the vacuum induction melting technique, homogenized at 1473 K for 5 h, and then quenched in water. Ingots with a thickness of 7.7 mm were hot rolled to 1 mm at 1373 K. In order to obtain fully recrystallized specimens with similar grain size, the rolled 2V, 6V, and 10V sheets were annealed at 1473 K for 90 min, 60 min, and 120 min, respectively.

Tensile specimens with a gauge length of 10 mm, width of 4 mm, and thickness of 1 mm were cut from annealed sheets by an electrical discharge machine, with the sample surface further polished to a 2000# grid. Tensile tests were carried out at an initial strain rate of 10^−3^ s^−1^ using a CMT 5105 testing machine at room temperature. A standard extensometer was used during the tensile test until fracture. The loading direction is parallel to the rolling direction (RD).

The crystal structure of the samples was characterized using a Smartlab (Rigaku, Tokyo, Japan) X-ray diffraction (XRD) device with a scanning angle of 20°–100° at a scanning rate of 2°/min. The FCC and HCP phases were indexed according to the XRD patterns of Co(γ) and Co(ε), respectively. Microstructural characterizations were conducted by JSM-7001F field emission scanning electron microscope (FE-SEM). Transmission electron microscope (TEM) characterization was conducted using JEM-2100F. The TEM samples were twin-jet electropolished using a solution mixture of 10% perchloric acid and 90% ethanol, with a direct voltage of 20 V and a temperature of 253 K.

## 3. Results

### 3.1. Microstructure of the Annealed Specimen

**Figure 1:**
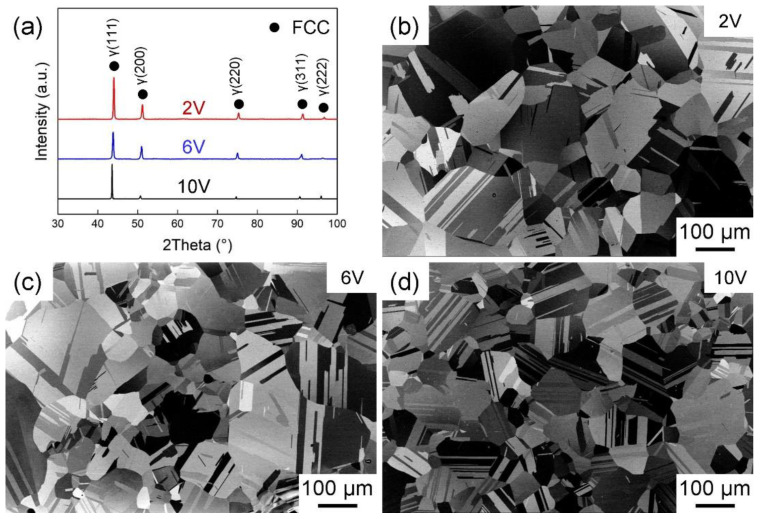
XRD patterns and SEM images of CoCrNiV alloys with different V contents. (**a**) XRD patterns of 2V, 6V and 10V. (**b**–**d**) SEM images of 2V, 6V and 10V.

The microstructure and XRD patterns of the annealed alloys with different V contents are shown in Figure 1.

The average grain sizes of the fully recrystallized alloys were measured by the line intercept method, including and excluding twin boundaries (TBs), respectively, and the statistical results are listed as 2V (37 ± 2 μm, 77 ± 2 μm), 6V (36 ± 4 μm, 68 ± 6 μm) and 10V (30 ± 2 μm, 57 ± 2 μm). All three alloys have similar grain sizes, excluding the grain size effect on the deformation mechanisms and mechanical properties. Further, the presence of annealing twins is captured in the majority of the grains. The XRD patterns show that the three fully recrystallized alloys are of FCC single-phase structure. The lattice constants of the three alloys were estimated from the XRD peaks and showed as follows: 2V (a = 0.3563 nm), 6V (a = 0.3576 nm), and 10V (a = 0.3580 nm), indicating a monotonous increase with V enrichment.

### 3.2. Mechanical Properties

**Figure 2:**
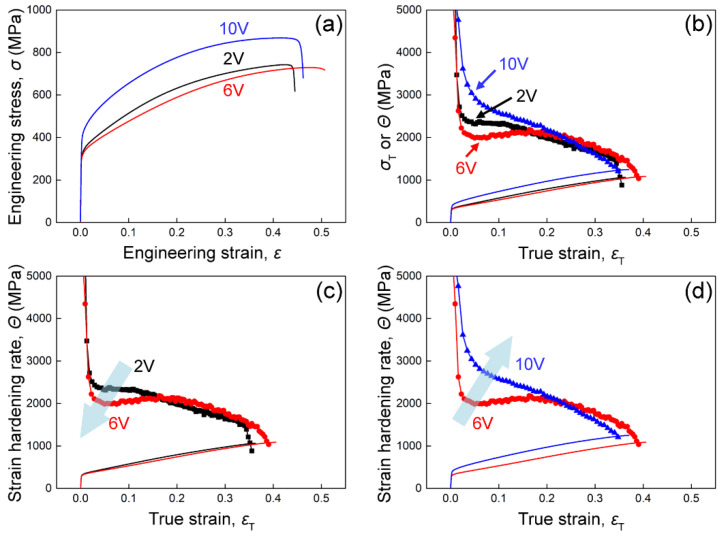
Mechanical properties of the CoCrNiV alloys: (**a**) engineering stress–strain curves; (**b**) true stress–strain curves and strain-hardening curves; (**c**) strain-hardening curves of 2V and 6V alloys; (**d**) strain-hardening curves of 6V and 10V alloys.

Figure 2 shows the tensile stress–strain curves and strain-hardening curves of the three alloys at room temperature. It is clear that the mechanical responses of the alloys are very sensitive to the V content. The 2V alloy has a yield strength of 318 MPa and a uniform elongation of 47%. With the increase in V content, the yield strength of 6V alloy decreases slightly to 307 MPa, and the uniform elongation increases to 49%. However, with the further increase in V content, the mechanical properties of the 10V alloy change dramatically. The yield strength of the 10V alloy increases to 415 MPa, and the uniform elongation decreases slightly to 42%. The strain-hardening rate is measured according to the true stress–strain curve (*Θ* = d*σ*/d*ε*). According to the Considére criterion, necking is predicted to occur when *Θ* = *σ* while the ductility of the alloy is largely related to its strain-hardening behavior [20]. Figure 2b shows the strain-hardening curves of the three alloys. The strain-hardening rate of the 2V alloy decreases rapidly first, then reaches a plateau and drops slowly until necking. As the V content increases to 6V, the strain-hardening curve of the alloy shifts down initially and surpasses the 2V alloy finally. Note that the strain-hardening rate can increase with strain, which generally is supposed to be induced by the TWIP effect [19]. However, this stage is induced by the dislocation planarity and stacking fault, which will be generally discussed in the following section. As the V content increases further to 10 at.%, the strain-hardening curve shifts up significantly. However, the strain-hardening rate rapidly decreases until necking. As shown in Figure 2c,d, this unusual change of strain-hardening curves with increasing V content is related to the variation of deformation mechanisms, which will be discussed in detail below.

### 3.3. Microstructure after Tensile Tests

**Figure 3:**
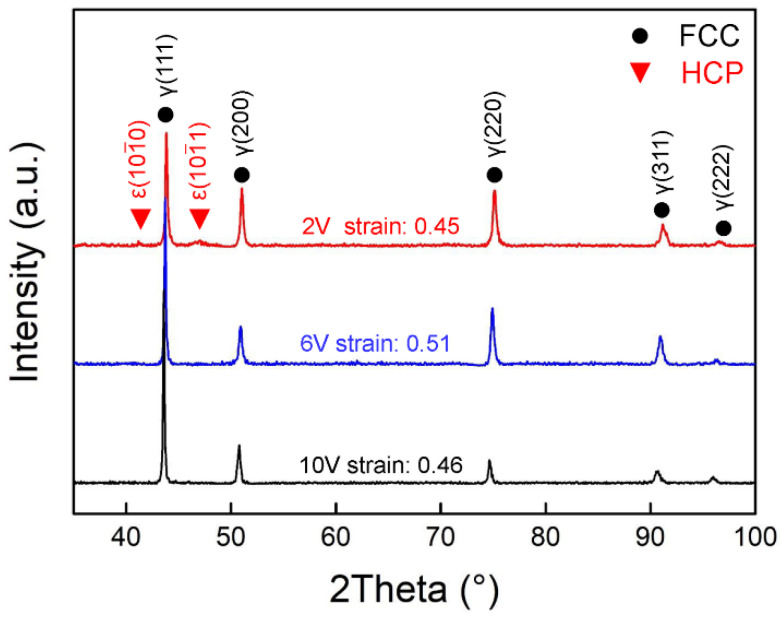
XRD patterns of the CoCrNiV alloys after tensile tests.

The accumulation of defects during deformation will lead to strain hardening of the alloys, such as dislocations, twins, and stacking faults (SFs) [21]. To reveal the reasons for the different strain-hardening behaviors of the three alloys, the deformation microstructures after tensile tests were characterized. Figure 3 shows the XRD patterns of the three alloys after tensile fracture, with samples taken from uniformly deformed parts of the gauge area. All three alloys had FCC single-phase structure before tensile tests (Figure 1a), but diffraction peaks of the HCP phase were detected in the 2V alloy after tensile fracture, indicating that the phase transformation was activated during tensile tests. This is in accordance with the deformation behavior of a non-equiatomic Co_36_Cr_41_Ni_23-_based alloy [19]. However, 6V and 10V alloys have FCC single-phase structures after deformation, showing high phase stability.

**Figure 4:**
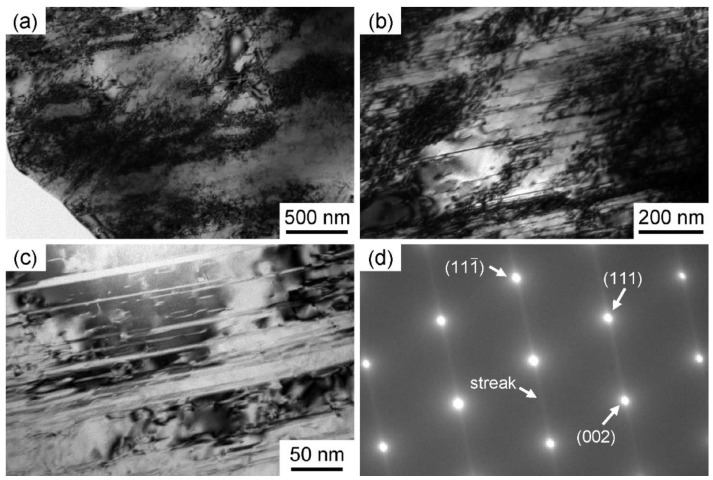
Deformation microstructures of 6V alloys after tensile tests. The SAED pattern in (**d**) is related to (**b**). (**a**–**c**) are the microstructures at different magnifications.

**Figure 5:**
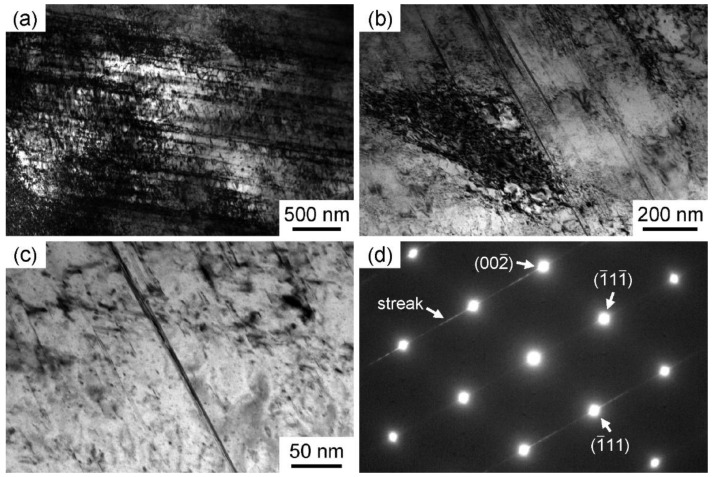
Deformation microstructures of 10V alloys after tensile tests. The SAED pattern in (**d**) is related to (**c**). (**a**–**c**) are the microstructures at different magnifications.

Since the substructure and phase transformation phenomena are closely related to different mechanical behaviors, TEM characterization after deformation was performed. In the TEM images of the deformation microstructures of the three alloys, the HCP phase and SFs all appear as lamella, which cannot be simply distinguished by TEM morphology. In contrast, the selected area electron diffraction (SAED) pattern is used to identify the deformation pattern. The SAED patterns of SFs and HCP phases are different. SF is a kind of planar defect. Under electron diffraction, the diffraction point of the faulted plane can be elongated along a specified direction in the reciprocal lattice, forming the streak pattern. Figure 4 and Figure 5 show the TEM images of the deformation microstructures of 6V and 10V alloys, respectively.

High-density dislocations and SFs were observed within the grains of both alloys, but deformation twinning and phase transformation were rarely detected. The reason for the deformation microstructure can be attributed to the severe lattice distortion of 6V and 10V alloys. For 6V and 10V alloys, according to the classical dislocation theory, severe lattice distortion causes atoms to deviate from equilibrium positions, which affects dislocation nucleation and motion [22,23]; that is, the full dislocation will be dissociated into Shockley partial dislocations to form SFs. However, deformation twinning and phase transformation were not activated. In contrast to 2V and 6V alloys, the main deformation mechanism of the 10V alloy is dislocation slip, in which the contribution of SFs decreases further. The strain-hardening curve of the 10V alloy continuously decreases, but the stage of increased strain-hardening rate in the 6V alloy is not observed, which is due to the increase in SFE of CoCrNi-based alloys with V addition. Note that in alloys with lower SFE, the lower V content favors the activation of SFs [24], which can retain the strain-hardening rate to high strains.

**Figure 6:**
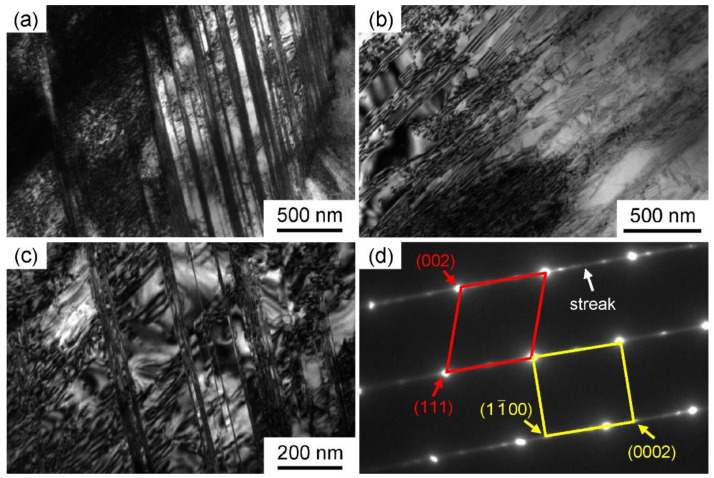
Deformation microstructures of 2V alloys after tensile tests. The SAED pattern in (**d**) is related to (**c**). (**a**–**c**) are the microstructures at different magnifications.

In contrast to the plastic deformation mechanism of 6V and 10V alloys, which is dominated by dislocations and SFs, the phase transformation is activated during the tensile test of 2V alloy, and its deformation microstructure is shown in Figure 6. Further high-density dislocations and SFs, thin HCP phase layers were detected. The SAED pattern in Figure 6d shows two sets of diffraction spots belonging to the HCP phase and the matrix. Based on the SAED pattern, the zone axis of the two phases has the relation of ${\left[110\right]}_{\mathrm{FCC}}//{\left[11\overset{-}{2}0\right]}_{\mathrm{HCP}}$. The activation of the fine HCP platelets at room temperature is mainly attributed to the extremely low SFE of the alloy, which can lead to the transition of the deformation mechanism from deformation twinning to deformation-induced martensitic transformation [19]. It can be indicated that the plastic deformation of 2V alloy at room temperature is dominated by dislocation slip, SFs, and deformation-induced martensitic transformation.

## 4. Discussion

### 4.1. Effect of V Addition on the SFE and Lattice Distortion

In random alloys, the disordered arrangement of solutes raises the interaction energy between dislocations and local solute concentration fluctuations. Dislocations are pinned in regions of favorable fluctuations, which are associated with high potential barriers to dislocation motion that can be overcome by applying high stresses and/or high temperatures. Yin et al. [15] found that V can improve the mechanical properties of FCC HEAs based on the theory of interaction energy between dislocations and solutes in random alloys. The addition of V to the Cantor alloy family was calculated to predict the large volume misfits that are more capable of producing high strength relative to other alloying elements, and this prediction was experimentally demonstrated in the CoCrFeMnNi alloy [25]. Based on the material parameters in the solid solution strengthening model for each alloy, the addition of V is capable of producing large atomic volume misfits in FCC alloys, and thus, V can be a suitable enhancing element in FCC HEAs [15]. V significantly increases the atomic spacing fluctuations, leading to structures that are significantly distorted relative to the ideal FCC lattice [14]. It is thus reasonable to add V into CoCrNi alloy to produce large atomic volume misfits, severe lattice distortion, and solid-solution strengthening effects. This drastic lattice distortion enhances lattice friction stress, which can be quantified by Peierls stress as expressed by the following equation:

*σ*_0_ = 2*MG*/(1 − *v*) · exp(−2π*w*/*b*) (1)

where *M* = 3.06 is the Taylor factor, *G* is the shear modulus, *v* is the Poisson’s ratio, *w* is the dislocation width, and *b* is the magnitude of the Burgers vector. The lattice friction stress strongly depends on the dislocation width and Burgers vector. The addition of V to CoCrNi alloys causes fluctuations in the atomic spacing, resulting in locally fluctuating Peierls’ potential for dislocation glide, increasing friction stresses [26]. Depending on the amount of V addition, the degrees of lattice distortion and, therefore, the solid-solution strengthening effect are different. The solution strengthening is closely related to the volume misfit; the calculated δ parameters of 2V, 6V, and 10V alloys are 1.96%, 2.33% and 2.61% [13]. When the V content is low (2V and 6V), the effect of solution strengthening is weak. The statistical grain sizes of 2V and 6V alloys seem similar, but the grain distribution of a specified specimen may change slightly, so the yield strength may change in a small range. This may explain the comparable yield strength of 2V and 6V alloys. When the V content increases to 10 at.%, the solution-strengthening effect becomes notable, so the yield strength of 10V alloy is significantly higher than 2V and 6V alloys. Chen et al. [24] calculated the SFE of V_0.5_Cr_0.5_CoNi alloy and referenced the calculated and experimental results of the SFE of CrCoNi and VCoNi alloys. They found that V addition to CoCrNi alloy could induce an increase in the SFE with the V content.

### 4.2. Non-Monotonic Change of the Strain-Hardening Behavior with V Addition

**Figure 7:**
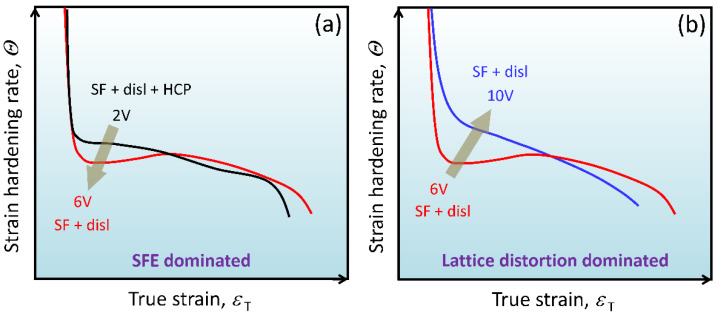
Schematic illustration of the deformation mechanisms and strain-hardening behavior of the alloys. (**a**) 2V and 6V alloys; (**b**) 6V and 10V alloys.

It is apparent that the strength and strain-hardening capability of the alloys changes considerably with V addition, as shown in Figure 2. However, the strain-hardening curve does not change monotonically as the V content increases. When the V content increases from 2 at.% to 6 at.%, the strain-hardening curve drops; in comparison, with the further increase of V content to 10 at.%, the strain-hardening rate rises sharply. Such differences can be related to the transition of the deformation mechanisms of the alloys. Figure 7 shows a schematic illustration of the strain-hardening behavior and related deformation mechanisms of the alloys, which will be discussed in detail below.

The strain-hardening rate of 2V alloys decreases rapidly at the beginning of deformation due to recovery. Subsequently, the strain-hardening rate levels off and then decreases slowly. This strain-hardening behavior can be related to the low SFE of the 2V alloy. Full dislocations are prone to dissociate into Shockley partial dislocations on {111} slip planes, forming SFs during the plastic deformation process [27]. These SFs are supposed to impede dislocation motion and induce a strong strain-hardening effect, retaining the high strain-hardening rate and forming the plateau. With a further increase in tensile strain, the local stress reaches the critical value for deformation-induced phase transformation, and thin HCP lamellae are activated [28,29], which can effectively impede dislocation motion [30]. During the deformation process, nano-sized HCP lamellae are alternately arranged, which relieves the local stress concentration of the alloy and delays the necking process [31]. These high-density dislocations, SFs, and HCP phases act synergistically to improve the strain-hardening ability of the alloy.

The SFE of the alloy increases with V addition. In this case, the HCP phase is not detected in the 6V and 10V alloys after tensile fracture. Meanwhile, the dislocation slip is affected, so the strain-hardening behavior can be affected by the V content. The strain-hardening rate of the 6V alloy is lower than that of the 2V alloy when the strain is smaller than 0.15, which can be a result of raised SFE. Note that the strain-hardening rate of the 6V alloy starts to increase when the strain is higher than 0.05. This phenomenon is usually considered to be caused by the TWIP effect. However, only dislocations and SFs are generated during plastic deformation, and there is no activation of deformation twinning. The increased strain-hardening rate can be attributed to the interaction between SFs and dislocations since SFs prevent the dislocations from gliding on the slip plane [32,33]. In other words, the increase in strain-hardening rate can be induced by SFs, which was also reported in Cu-Al alloys [10]. While in the final stage of deformation, it is increasingly hard to introduce dislocations and SFs; thus, the strain-hardening rate decreases until fracture. When the V content is further increased to 10 at.%, the strain-hardening curve moves upwards, as shown in Figure 7b. It is noteworthy that the yield strength and strain-hardening rate of 10V alloy are higher than 2V and 6V alloys. Since the SFE of the 10V alloy is the highest among the three alloys, the enhanced strain-hardening capability may result from different mechanisms, that is, the severe lattice distortion. The high V content can significantly increase atomic spacing fluctuations, leading to severe lattice distortion and increased lattice friction stress and yield strength [26]. Severe lattice distortion can facilitate the dissociation of full dislocations to form SFs, serving as barriers for dislocation slip. In this case, it is believed that lattice distortion instead of SFE dominates the strong strain-hardening behavior of 10V alloy.

Above all, the addition of V into the CoCrNi alloy influences the SFE and lattice distortion of the alloy. As the V content increases, on the one hand, the SFE of the alloy increases, making it difficult to activate SFs and phase transformations; on the other hand, the lattice distortion becomes severe, which retards the dislocation slip. In general, the SFE of 2V alloy is low, and its deformation mechanisms are mainly dislocations, SFs, and phase transformation; the SFE of 6V and 10V alloys increase, and their deformation mechanisms are mainly dislocations and SFs. The opposite change of strain-hardening curves in Figure 7 can be a result of SFE and lattice distortion, which were strongly related to the V content.

## 5. Conclusions

Different V alloyed (named 2V, 6V, and 10V) non-equiatomic CoCrNi alloys were selected to investigate the tensile properties and plastic deformation mechanisms. The main conclusions can be summarized as follows:

1. Singe-phase FCC CoCrNiV alloys with different V additions were obtained after solid solution treatment.
2. The plastic deformation of 2V alloy is dominated by dislocation slip, SFs, and HCP martensitic transformation. With increasing the V content, the SFE of CoCrNiV alloy increases, and dislocation slip and SFs prevail in 6V and 10V alloys.
3. The strain-hardening behavior changes non-monotonically with increasing the V content. The lower strain-hardening rate of 6V alloy than that of 2V alloy is dominated by the SFE. The higher strain-hardening rate of 10V alloy than that of 6V alloy is dominated by the lattice distortion.

## Data Availability

The data presented in this study are available on request from the corresponding author.
